# Convulsions

**Published:** 1846-02

**Authors:** 


*Convulsions.*—Recent evidence in children, shows that the ratio is in proportion to the standard of unhealthiness. Thus, on an average of the three years, 1838–'40, the proportion of deaths by convulsions and teething to the total deaths from *all* diseases was, in Birmingham 5·70 per cent., in London 7·30, in Leeds 12·20, in Manchester 13·70, and in Liverpool 15.—*Ibid.*

